# The relationship between stress and vitiligo: Evaluating perceived stress and electronic medical record data

**DOI:** 10.1371/journal.pone.0227909

**Published:** 2020-01-27

**Authors:** Steven W. Henning, Dinesh Jaishankar, Levi W. Barse, Emilia R. Dellacecca, Nicola Lancki, Kirsten Webb, Linda Janusek, Herbert L. Mathews, Ronald N. Price, I. Caroline Le Poole

**Affiliations:** 1 Oncology Research Institute, Loyola University Chicago, Maywood, Illinois, United States of America; 2 Department of Dermatology, Feinberg School of Medicine, Northwestern University, Chicago, Illinois, United States of America; 3 Department of Preventive Medicine, Northwestern University, Chicago, Illinois, United States of America; 4 Division of Dermatology, Loyola University Chicago, Maywood, Illinois, United States of America; 5 Marcella Niehoff School of Nursing, Loyola University Chicago, Maywood, Illinois, United States of America; 6 Department of Microbiology and Immunology, Loyola University Chicago, Maywood, Illinois, United States of America; 7 Office of Informatics and System Development, Loyola University Chicago, Maywood, Illinois, United States of America; 8 Department of Microbiology and Immunology, Feinberg School of Medicine, Northwestern University, Chicago, Illinois, United States of America; Kinki Daigaku, JAPAN

## Abstract

Vitiligo is a T-cell mediated skin disorder characterized by progressive loss of skin color. In individuals genetically predisposed to the disease, various triggers contribute to the initiation of vitiligo. Precipitating factors can stress the skin, leading to T-cell activation and recruitment. Though hereditary factors are implicated in the pathogenesis of vitiligo, it is unknown whether precipitating, stressful events play a role in vitiligo. To understand this, we utilized a validated perceived stress scale (PSS) to measure this parameter in vitiligo patients compared to persons without vitiligo. Additionally, we probed a clinical database, using a knowledge linking software called ROCKET, to gauge stress-related conditions in the vitiligo patient population. From a pool of patients in an existing database, a hundred individuals with vitiligo and twenty-five age- and sex-matched comparison group of individuals without vitiligo completed an online survey to quantify their levels of perceived stress. In parallel, patients described specifics of their disease condition, including the affected body sites, the extent, duration and activity of their vitiligo. Perceived stress was significantly higher among vitiligo individuals compared to those without vitiligo. ROCKET analyses suggested signs of metabolic-related disease (i.e., ‘stress’) preceding vitiligo development. No correlation was found between perceived stress and the stage or the extent of disease, suggesting that elevated stress may not be a consequence of pigment loss alone. The data provide further support for stress as a precipitating factor in vitiligo development.

## Introduction

Vitiligo is an acquired skin disorder characterized by a progressive loss in skin pigmentation due to the loss of melanocytes, the pigment producing cells in the skin. Among other tissues, melanocytes are present in the inner ear, eye and mucosal membranes [1–3] and, are therefore, also affected due to vitiligo [4]. Vitiligo affects around 0.5% of the global population and, although all ethnic groups are similarly affected, it is more noticeable and more severe in dark-skinned individuals [5, 6]. While the onset of vitiligo usually occurs during adolescence [6], individuals developing vitiligo during adulthood have been reported [7, 8]. Though hereditary factors predispose patients to depigmentation, in adult onset vitiligo, a relatively greater contribution to disease etiology can be attributed to stress [9, 10].

Vitiligo predisposition is defined by variant sequences at loci associated with both the innate and adaptive immune system as well as to loci associated with melanogenesis and apoptosis [11–15]. Precipitating factors have been acknowledged, including exposure to sunlight or skin trauma, leading to oxidative stress in melanocytes [16–19] and T-cell mediated autoimmune responses [20, 21]. Indeed, while vitiligo has been established as an autoimmune entity, [21], the mechanism connecting the initiating event(s) to the induction of anti-melanocyte T-cell immunity is unknown.

Physical or environmental stressors are reported in the onset and disease progression of vitiligo [22–24]. In the event of a sunburn or exposure to certain chemicals or skin trauma, free radicals and hydrogen peroxide are generated [25], and in individuals who are predisposed to vitiligo, this leads to an activation of the immune system that generates melanocyte-specific cytotoxic responses. Heat shock proteins (HSP) are cellular stress response proteins that protect a cell under stressful conditions [26]. Notably, among the family of HSP, inducible HSP70 (HSP70i) is secreted by live cells under stress [27]. Previous work from our lab showed a critical role of HSP70i from the melanocytes in accelerating autoimmune vitiligo [28, 29]. Stressed cells secrete HSP70i and in the extracellular milieu, HSP70i can activate dendritic cells (DCs) and aid in antigen cross-presentation [30], resulting in cytotoxic T cell responses to melanocytic antigens.

Psychological stressors also play a role in vitiligo [23, 24]. Events such as death of a family member, work and financial problems have been associated as preceding factors to the onset of vitiligo [24]. In addition, vitiligo patients experience severe psychological effects [31, 32] and exhibit anxiety [33], depression [13], social stigma [34] and impaired quality of life [35, 36]. Stress increases the levels of catecholamines, neuropeptides, and cortisol that are higher in vitiligo patients [37–39] suggesting their role in the pathogenesis of vitiligo.

To understand the association of stress in vitiligo patients, in this study, we used a validated questionnaire [40] to assess levels of perceived stress (PSS) [41] in vitiligo and healthy age- and gender- matched individuals. Patients were asked some additional questions about their condition, and ROCKET software was used to probe a patient database to explore the prevalence and chronology of stress related conditions among vitiligo patients. The data serve to correlate stress and vitiligo, providing support for the concept that stress can influence progressive depigmentation of the skin.

## Methods

### Sample size

One-hundred vitiligo patients who previously emailed us about our vitiligo research efforts were contacted and invited to participate in this study. The study was approved by the Loyola University Chicago Institutional Review Board. They were also asked to identify a person in their direct environment (not a blood relative) of the same sex and of the same age +/- 5 years. Though some patients provided personal contact information, the questionnaire responses could not be linked to the submitter.

### Vitiligo questionnaire

A previously published questionnaire was provided to the patients to assess and record their disease etiology [42]. Patients diagnosed with vitiligo by a physician were requested to answer questions related to their condition including their sex, disease activity, and lesional distribution. Data are shown in S1 Table.

### Perceived stress scale (PSS) and scoring

The PSS is a 10-item scale, which asks participants to rate the degree to which life experiences over the past 30 days are perceived as uncontrollable. PSS is a widely used measure of general perceived stress [40]. Reliability (stability) is 0.85 and Cronbach alphas range from 0.75–0.86 [43]. The threshold for stressed individuals is set to a PSS score of 15 as described [44]. Data are shown in S1 Table.

### Questionnaire administration

The vitiligo and PSS questionnaires were electronically uploaded to Google Drive. Study participants were given access to the Drive to record their own responses, while maintaining anonymity.

### Relationship of clinical knowledge and events tool (ROCKET)

ROCKET software can be used to probe the Clinical Research Database or ‘CDRB’ to query a limited dataset (LDS) repository of electronic patient records without personal identifiers, covering about 9 million encounters between 1/1/2007 and 9/30/2015. The software allows the investigator to locate information related to a particular patient population (here: vitiligo patients) and compare outcomes to those among unaffected controls. All patients at all encounter types where an ICD9 code of 709.1 or an ICD10 code of L80 was assigned are included. This covers a target population of just over 1000 subjects. As psychological stress and metabolic syndrome (metabolic stress) are associated with vitiligo [45–47], anemia and depression (markers for psychological stress) and hypertension and hyperlipidemia (markers for metabolic stress [48]) conditions were probed. The requested information can include demographics, encounter information, order codes, chronic disease status and calculation of comorbidities, medication and clinical lab results.

### Statistical analysis

PSS among healthy and vitiligo patients, and among male and female vitiligo patients were compared. The responses from the vitiligo questionnaire were correlated to outcomes from the PSS questionnaire. Unadjusted odds ratios (OR), a measure of association between an exposure and an outcome, and 95% confidence intervals (CI) were computed for stress-related conditions in the vitiligo group compared to the general population group. A paired t-test or Mann-Whitney U-test was used to determine differences between two groups, whereas ANOVA was used to determine differences among three or more groups. Pearson’s correlation coefficients were computed to determine associations between continuous variables. Statistical analysis was performed using GraphPad Prism Software (V8), and the odds ratio were computed using STATA.

## Results

### Vitiligo patients experience an increase in perceived stress

**Fig 1:**
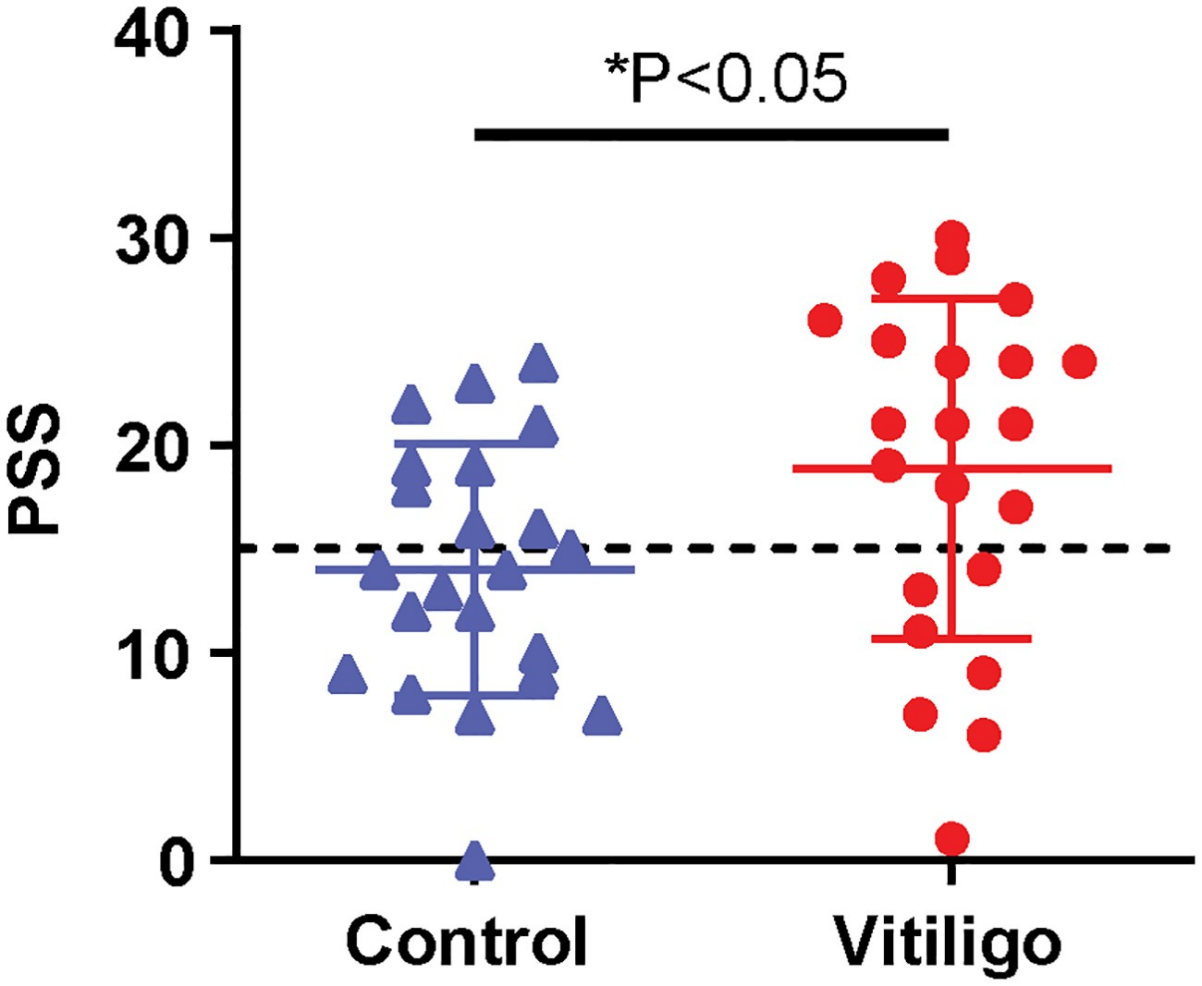
Perceived stress is higher in vitiligo patients. PSS scores of healthy versus and vitiligo age-matched patients were compared. Sample includes both males and females (n = 25). The dashed line indicates the cutoff score for stressed individuals (at PSS = 15). Statistical significance for PSS among the populations was determined by paired two-tailed t-test, P = 0.0396.

We hypothesized that vitiligo patients experience more stress than individuals without vitiligo. Among 102 participating patients, 54.8% were male and 45.2% were female. At the time of completion of the PSS, 63.5% of patients described their disease activity as active, while 31.7% described their disease as stable disease and the remainder (4.8%) reported regressing disease. As patients are most distressed about vitiligo developing in the more visible, sun-exposed areas of their skin (unpublished), we tallied vitiligo development in different body parts. Among participants, 75% or more of patients developed vitiligo of their hands and of their face, which is commonly perceived as the most impactful by patients. Other commonly visible body parts include the extremities, which affected 50% or more of the sample at the time of data collection. The mean PSS score for vitiligo patients was 19.3, whereas, age and sex-matched controls had a mean PSS score of 13.8 (Fig 1, n = 22; P = 0.0396 in a paired t-test). A 2009 US probability sampling of adults documented a mean PSS score of 15.2 [44], suggesting that the present cohort of vitiligo patients had elevated PSS scores. That is, they perceived events in their life to be less manageable. We thus probed the full population of participating vitiligo patients for a more in-depth evaluation of levels of perceived stress.

### Female vitiligo patients perceive more stress than affected males

**Fig 2:**
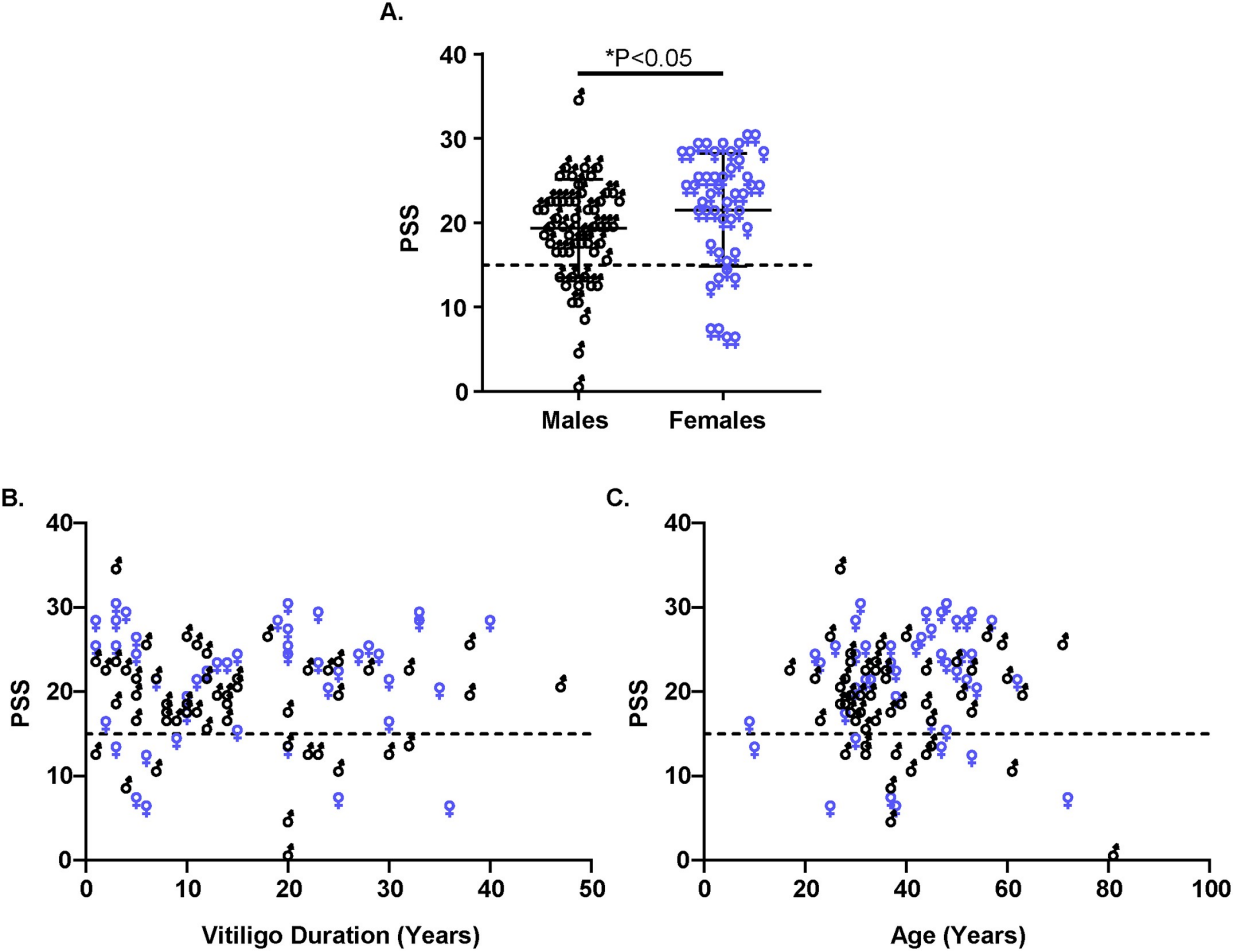
Perceived stress is higher among female vitiligo patients. **(A)** PSS scores were compared between male (n = 56) and female (n = 47) vitiligo patients. Statistical significance was determined by Mann-Whitney test, P = 0.0143. **(B-C)** Scatter plot illustrates relationship between PSS scores and vitiligo duration (B, P = 0.7955); and between PSS scores and age (C, P = 0.8746). (B) and (C) include both male and female vitiligo patients. Dashed line indicates the PSS cutoff score for stressed individuals (at PSS = 15).

Among all vitiligo participants, we compared the PSS scores between males and females (n = 55 and 47, respectively). While both groups reported a mean PSS score indicating stressed population (Fig 2A, PSS males 19.4 and PSS females 21.53), female patients perceived significantly more stress compared to male patients (P = 0.0143). An evaluation of the relationship between PSS scores per group relative to either duration of vitiligo (Fig 2B) or age (Fig 2C), revealed no significant correlations in either male or female patients.

### Perceived stress is not related to disease activity

**Fig 3:**
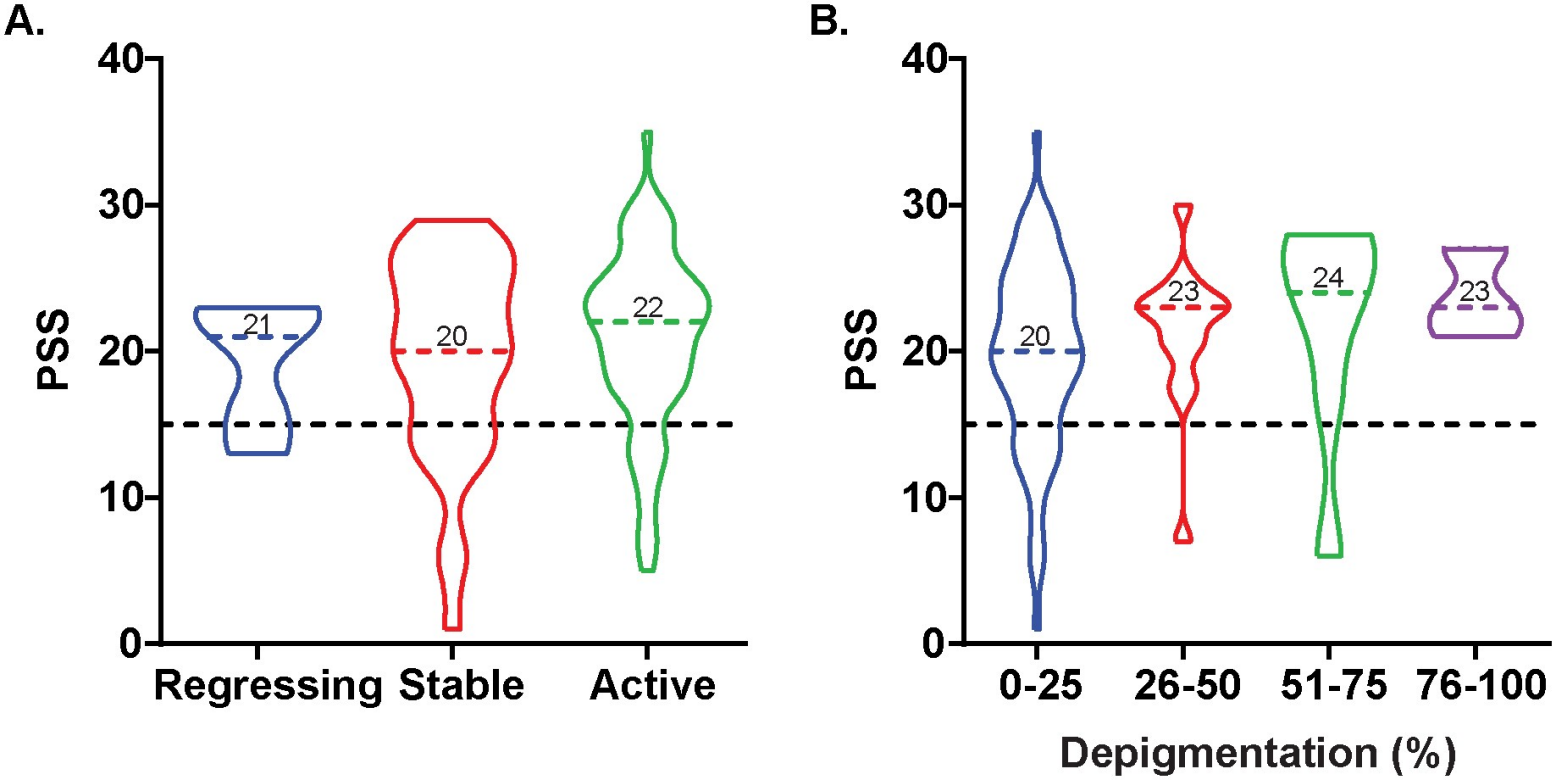
Perceived stress is not associated with disease progression and self-reported depigmentation. **(A)** Violin plots depicting the distribution of PSS among the three stages of vitiligo progression. Colored dashed line and number indicate the median values. **(B)** Violin plots depicting the distribution of PSS in the four categories of self-reported depigmentation. Dashed line indicates the PSS cutoff score for stressed individuals (at PSS = 15).

Almost two-thirds of patients evaluated were in an active phase of their disease, with others experiencing disease stability or regression. There was no difference in PSS values among patients experiencing differences in disease activity (Fig 3A); thus, we next examined the possibility as to whether perceived stress may be associated with self-reported extent of depigmentation (Fig 3B). The percentage of self-reported depigmentation was divided into four categories, based on percent of depigmentation (0–25; 25–60; 51–75; and 76–100). Although no significant correlation was observed between percentage of depigmentation and PSS scores, interestingly, a wide distribution of PSS among the 0–25% depigmentation group was observed. This figure also demonstrates that PSS scores are not related to the age of participating patients. As vitiligo development is commonly believed to initiate most frequently in the second decade of life, this would suggest that patient age might reflect the duration of disease. To determine if this is the case, we probed the anonymized clinical database available at Loyola using ROCKET software.

### Vitiligo is primarily diagnosed between two distinct age groups

**Fig 4:**
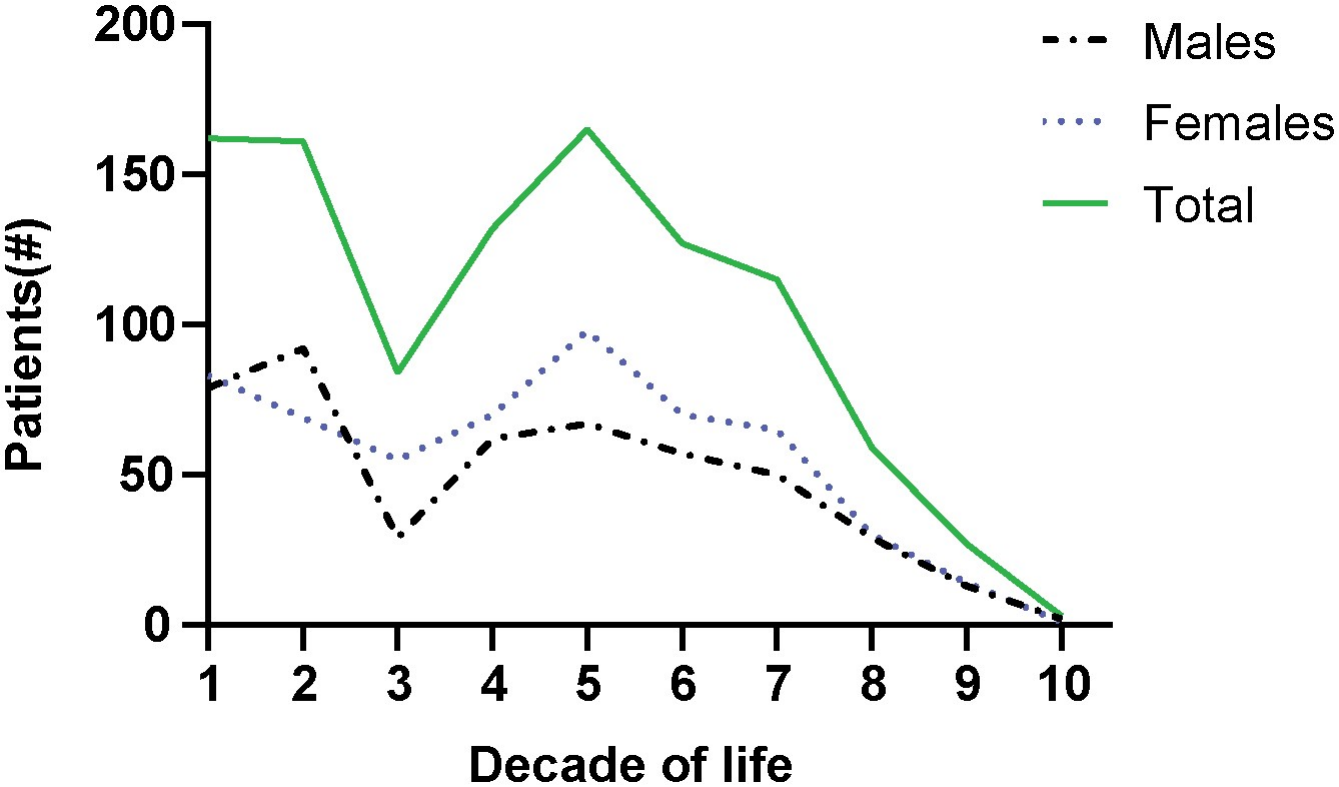
Bi-phasic age groups of vitiligo diagnosis. The ROCKET database was probed to determine the number of patients diagnosed with vitiligo. Total, male and female vitiligo populations are shown.

To understand how the timing of other diagnoses and treatment relate to vitiligo development, we probed and plotted the age at diagnosis among vitiligo patients in the ROCKET database (Fig 4). The results revealed a biphasic pattern, the first one covering the first 2 decades of life peaking slightly later among males, than among females and the second peaking in the 5^th^ decade of life. This is not dependent on the number of patients registered in the system for every age group, as the biphasic peaks were not observed for other conditions (unpublished). Instead, the distinct peaks would suggest that different etiologic factors prevail in different phases of life. This provided a framework to use the ROCKET database to probe other parameters among vitiligo patients.

### The timing of metabolic stress and not psychological stress can support a causative relationship among vitiligo patients

**Fig 5:**
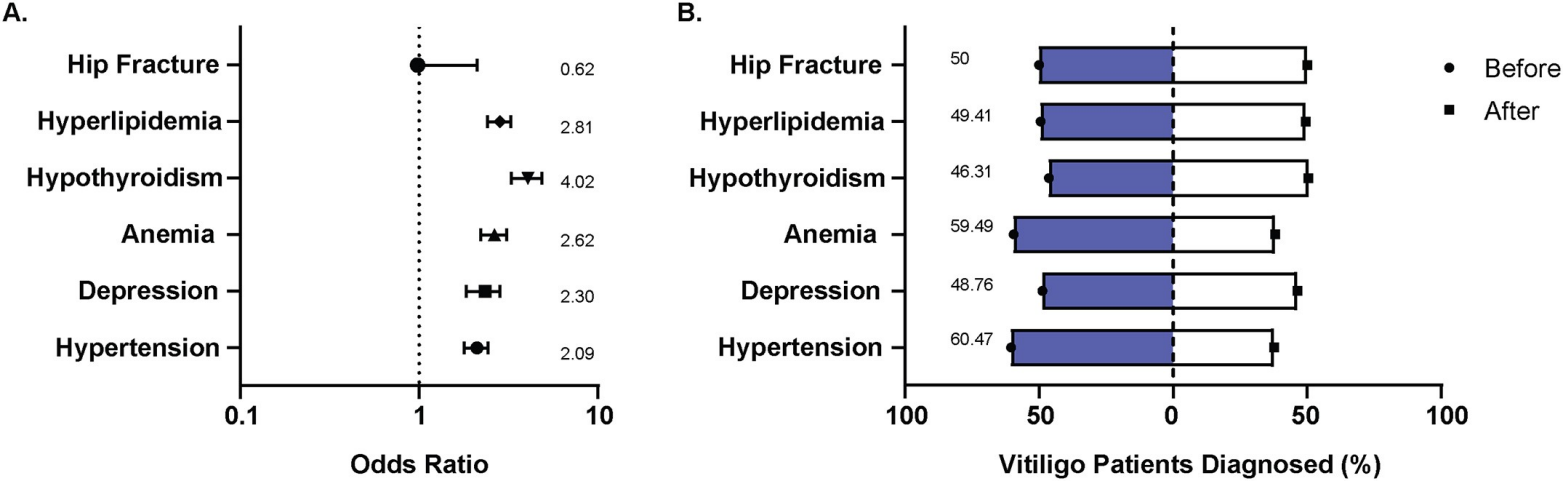
Prevalence and timing of metabolic stress parameters among vitiligo patients. **(A)** The odds of expressing a comorbidity among vitiligo patients, compared to the general patient population expressed as an odds ratio. A value of 1 indicates odds equal to the general patient population. Values greater than 1 indicates greater, and lower than 1 indicates smaller odds. The error bars indicate the upper and lower confidence intervals **(B)** Timing of diagnosis for indicated comorbidities compared to the diagnosis of vitiligo among patients.

Hypothyroidism is the most common autoimmune disorder associated with vitiligo [49, 50]. To confirm this, we initially probed the database to determine whether hypothyroidism is increasingly prevalent among vitiligo patients (not as a marker for metabolic stress). Consistent with previous literature, we observed that the percentage of patients (11.69% in vitiligo vs 3.19% in general) and the odds (OR 4.02, CI 3.29 to 4.88) of hypothyroidism diagnosis is higher among vitiligo patients (Fig 5A). We then probed the database for the prevalence of hypertension and hyperlipidemia among vitiligo patients compared to the general patient population as a possible sign of metabolic stress among patients [51]. Indeed, the percentage of being diagnosed with hypertension (20.30%; OR 2.09, CI 1.79 to 2.44) and hyperlipidemia (22.90%; OR 2.81, CI 2.42 to 3.26) were higher among vitiligo patients (Fig 5A). Upon probing the timing of diagnosis, more patients were diagnosed with these metabolic disorders prior to their vitiligo diagnosis than after, suggesting a causative factor for vitiligo (Fig 5B). As a possible sign of psychological stress, the database was probed for patients diagnosed with depression and anemia [52–55]. Interestingly, while the percentage of patients and the odds of being diagnosed with depression (9.18%; OR 2.3, CI 1.84 to 2.85) and anemia (16.62%; OR 2.62, CI 2.22 to 3.1) were high among vitiligo patients (Fig 5A), the timing prior to and after vitiligo showed a similar percentage of patients diagnosed (Fig 5B). This would suggest that both conditions might involve common etiologic factors.

**Fig 6:**
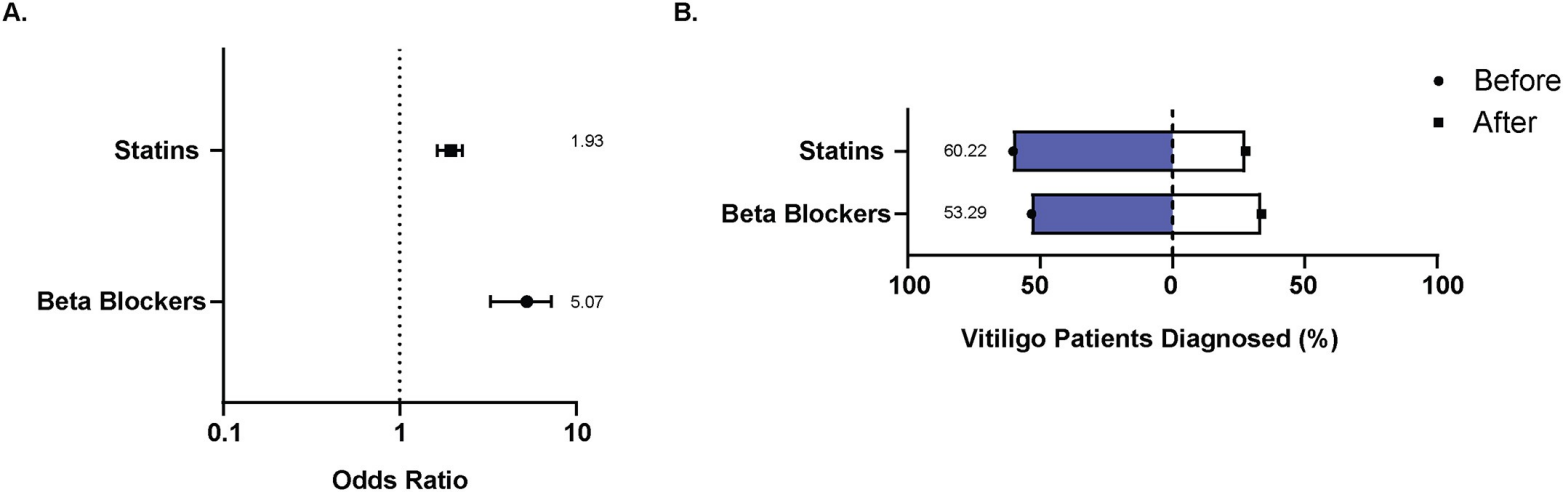
Prevalence and timing of prescription drugs for metabolic stress. **(A)** Odds ratio depicting the odds of being prescribing statins of beta blockers among vitiligo patients compared to the general patient population. The error bars indicate upper and lower confidence intervals. **(B)** Timing of first drug prescription compared to the timing a vitiligo diagnosis was made among patients.

To further support the role of metabolic stress in vitiligo, we probed the database for patients prescribed beta-blockers and statins [56, 57]. In line with our above findings, we observed that the percentage of patients and the odds of statins (17.49%; OR 1.93, CI 1.64 to 2.27) and beta-blockers (16.14%, OR 5.07, CI 3.37 to 7.33) prescription were higher among vitiligo patients (Fig 6A). In addition, more patients were prescribed the drugs prior to their vitiligo diagnosis, than after (Fig 6B). These differences are even more impressive when accounting for patient age at vitiligo diagnosis (Fig 4). Taken together, these results indicate that signs of (metabolic) stress are more frequently observed among vitiligo patients, and largely precede disease diagnosis. Thus, patients in part develop disease following signs of stress, producing a dataset informative of a potentially causative relationship.

## Discussion

To understand whether vitiligo associates with stress, the PSS and ROCKET analyses in our study newly revealed that ‘metabolic’ stress precedes and might thus contribute to vitiligo. Indeed this is in line with studies suggesting environmental and psychological stressors are triggers for the onset and progression of vitiligo [24]. Although the exact mechanism(s) by which stress influences vitiligo remains unknown, as discussed earlier, both environmental and psychological stress result in autoimmune vitiligo [21].

The PSS questionnaire is a validated tool to measure perceived stress and several studies have used this tool to estimate perceived stress in patients, including those with autoimmune disease [58, 59]. Among the vitiligo patients who participated in this study, female patients perceived significantly more stress than male patients did. Other studies have also found female patients to be increasingly impacted by stress [22, 31]. Whether women are more conscious of their stress or there are other underlying factors that attribute to their perception remains uncertain. In vitiligo, the depigmentation itself can also cause stress and in fact, self-reported depigmentation was the highest on the face and hands (S1B Fig) supporting this former notion. In the current study, neither age, nor duration of vitiligo or disease status were associated with perceived stress. PSS only measures a person’s perception of stress over the past month. It does not capture cumulative stress and it does not capture the quantity of negative life events. Thus, this limits the ability to make any inferences as to the role of cumulative life stress, which is more likely to influence vitiligo development and/or progression. It is also likely that social support and adaptive coping may buffer the impact of stress associated with vitiligo, which may explain why levels of perceived stress did not associate with vitiligo duration or characteristics.

Stress and stressors can have a profound impact on autoimmunity [60]. The timing and release of stress hormones regulate the pro- and anti-inflammatory cytokine balance that dictate immunoprotective or immunosuppressive activity [61, 62]. Acute or short-term stress results in an immunoprotective environment whereas chronic or long-term stress commonly result in an immunosuppressive environment [63]. Chronic stressors can, however, also promote a proinflammatory environment, resulting in dysregulated immune responses that might lead to autoimmunity [63, 64]. A limitation in this study is that no biochemical assays or cytokine profiles were investigated for these patients to correlate them to the PSS or vitiligo questionnaire. However, perceived stress did not correlate with disease state or duration. As stress hormones are increased in vitiligo patients [37, 38] and a cytokine imbalance has been assigned to oxidative stress in melanocytes, the data collectively prompt studies of a role for chronic stress in disease development.

The ROCKET analyses revealed a bimodal age of vitiligo diagnosis with the first age group peaking around 10–20 years and the second age group peaking around 50–60 years. First presented by us at the International Pigment Cell and Melanoma Research Conference in 2017, a recent GWAS study has since solidified this finding regarding vitiligo onset [65]. Diagnosis occurring at two different phases of life could implicate different etiological factors. Frequency of a stressful event was higher among adult patients compared to childhood onset [9], suggesting that stress is a precipitating factor particularly for adult onset vitiligo. In fact, the death of a loved one and work/financial problems are the most common stressful life events reported among adult vitiligo patients, and such events are consistently associated with a poor quality of life and depression [13, 66]. While the percentage of population affected and the odds of having depression were higher among vitiligo patients, the percentage of patients that were diagnosed with depression prior to and after vitiligo were similar in our study. This suggests that depression is neither a causative factor nor a consequence of vitiligo, but rather these conditions may share a common etiological factor. Metabolic syndrome is a cluster of disorders presenting with aberrant metabolism resulting in an increased risk of cardiovascular disease [51]. Chronic stress and stressful events present with a high risk of metabolic syndrome [67–69], and vitiligo patients are at a higher risk of developing metabolic syndrome [46, 70]. Consistent with previous findings, the ROCKET analyses revealed that the percentage of patients and the odds of developing metabolic disorders, hypertension and hyperlipidemia, were higher among vitiligo patients (Fig 5), and these disorders are linked to oxidative stress and autoimmunity [71–75]. Similarly, the odds for being prescribed statins and beta-blockers for cardiovascular disease, the major risk for metabolic syndrome, were higher for vitiligo patients. Taking into account the timing of diagnosis, patients were more frequently diagnosed with hypertension and hyperlipidemia, and more frequently so prior to their vitiligo diagnosis. This was accompanied by a greater percentage of patients prescribed statins and beta-blockers prior to their vitiligo diagnosis. A limitation of the current ROCKET analyses was that only a subset of comorbidities were available for analysis of the vitiligo patient cohort. Further, lifestyle factors, such as health behaviors, are important factors that contribute to the chronic diseases we probed, and must be considered in future studies investigating any associations of these diseases with onset and progression of vitiligo. Collectively, our data suggest that metabolic stress might be involved with the onset and progression of vitiligo. This prompts further analysis, including measurement of physiologic parameters.

In conclusion, the findings from this study indicate that vitiligo patients have high levels of perceived stress. In patients predisposed to vitiligo, metabolic and psychological stress might influence the onset and progression of vitiligo.

## Supporting information

S1 Fig Self-reported depigmentation. **(A)** Pie chart depicting the number of vitiligo patients with self-reported percentage depigmentation. Self-reported percentage depigmentation was categorized as 0–20, 21–40, 41–60 and 61–100% with each group having n = 70, 20, 6 and 8 vitiligo patients respectively. **(B)** Self-reported percentage depigmentation among different body parts.
(DOCX)

S1 Table (XLSX)
