# Supplementary material for: The relationship between stress and vitiligo: Evaluating perceived stress and electronic medical record data
Source: PLoS One. 2020 Jan 27;15(1):e0227909. doi: 10.1371/journal.pone.0227909 (PMC6984686; doi:10.1371/journal.pone.0227909)
Supplement: S1 Fig — (A) Pie chart depicting the number of vitiligo patients with self-reported percentage depigmentation. Self-reported percentage depigmentation was categorized as 0–20, 21–40, 41–60 and 61–100% with each group having n = 70, 20, 6 and 8 vitiligo patients respectively. (B) Self-reported percentage depigmentation among different body parts. (DOCX) [file pone.0227909.s001.docx]

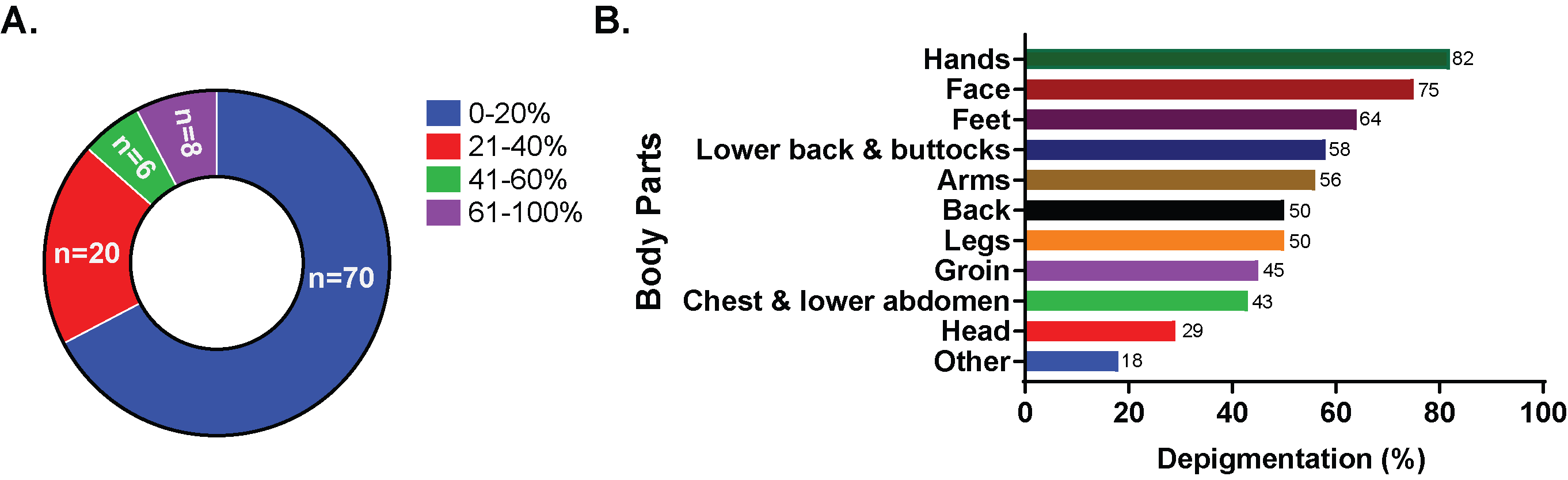


**Figure S1: Self-reported depigmentation. (A)** Pie chart depicting the number of vitiligo patients with self-reported percentage depigmentation. Self-reported percentage depigmentation was categorized as 0-20, 21-40, 41-60 and 61-100% with each group having n=70, 20, 6 and 8 vitiligo patients respectively. **(B)** Self-reported percentage depigmentation among different body parts.
